# Healthcare transition readiness of families of youth with cystic fibrosis during COVID-19: A correlational multicenter analysis

**DOI:** 10.1016/j.hctj.2024.100065

**Published:** 2024-07-26

**Authors:** Tyra C. Girdwood, Kirsten Kainz, Susan G. Silva, Jennifer L. Goralski, Maria E.Díaz-González de Ferris, Mary R. Lynn, Elisabeth P. Dellon, Samya Z. Nasr, Ahmet Uluer, Mark P. Toles

**Affiliations:** a University of North Carolina at Chapel Hill, School of Nursing, 120 North Medical Dr., Chapel Hill, NC 27599, USA; b Duke University, School of Nursing, 307 Trent Dr, Durham, NC 27710, USA; c University of North Carolina at Chapel Hill, School of Medicine, 321 South Columbia St., Chapel Hill, NC 27514, USA; d University of North Carolina at Chapel Hill, School of Social Work, 325 Pittsboro St Ste 3550, Chapel Hill, NC 27516, USA; e University of Michigan, Michigan Medicine, 1500 E Medical Center Dr, Ann Arbor, MI 48109, USA; f Harvard Medical School, Boston Children’s Hospital, 300 Longwood Avenue, Boston, MA 02115, USA

**Keywords:** Cystic Fibrosis, Healthcare Transition Readiness, Mental Health, Resilience, Adolescents and Young Adults, COVID-19

## Abstract

**Background:**

Enhancing family readiness for the healthcare transition (HCT) to adult-focused care can help adolescents and young adults (AYA) thrive in adulthood. We aimed to explore modifiable and non-modifiable individual, family, and healthcare factors associated with HCT readiness of AYA among families of AYA with cystic fibrosis (CF) during COVID-19.

**Methods:**

A multi-site, cross-sectional design was used and an online survey was deployed among families and their AYA from three US pediatric CF centers. The STAR_x_ Transition Readiness Questionnaire assessed caregiver- and AYA-perceived HCT readiness of AYA.

**Results:**

Caregivers (*N* = 71) and their AYA with CF (*N* = 33, aged 12–21 years) perceived moderately high HCT readiness of AYA. Caregiver resilience was a significant (*p* = 0.006), family-level factor correlated with caregiver-perceived HCT readiness.

**Conclusions:**

Families perceived similar levels of AYA readiness for adult-focused care. Caregiver resilience is an important, modifiable family-level factor for targeted interdisciplinary interventions aimed at enhancing HCT processes during COVID-19.

## Highlights

• Caregivers & AYA perceived similar levels of AYA healthcare transition readiness.
• Caregiver resilience was associated with caregiver-perceived readiness of AYA.
• COVID-19 changes may impact mental health and healthcare transition satisfaction.
• Modifiable family-level factors should be regularly assessed in clinical settings.

## 1 Introduction

In 2022, an estimated 5.6 million adolescents and young adults (AYA) with special health care needs like cystic fibrosis (CF) did not receive services necessary to transition from pediatric to adult-focused healthcare in the USA.^1^ Transition services, like providers working with families to build AYA self-management and advocacy skills, are important to enhance healthcare transition readiness (HCT) as AYA with CF experience longer life expectancies and will need skills to navigate changes in lifestyles, caregiver/patient care management, and coordination among providers.^2^ In 2022, approximately 13,244 (40.6 %) of the 32,621 individuals with CF in the USA were ≤ 17 years old.^3^ Hence, a critical CF subpopulation will need HCT readiness skills for enhanced self-care, disease knowledge, and provider communication to optimize outcomes in adulthood.

Compared to their peers, families of AYA with CF are at risk for gaps in HCT readiness due to high treatment burden, the progressive, multi-organ impact of their condition, and higher than average prevalence of depression and anxiety among caregivers and AYA.^2^, ^4^, ^5^ Poor HCT readiness may exacerbate risk of nonadherence, hospital admissions, and early mortality in adulthood.^5^, ^6^, ^7^ Emerging evidence suggests caregiver education and employment, AYA age (older patients), sex (females), and two-caregiver households are associated with HCT readiness.^6^, ^7^ However, an important gap is the identification of modifiable and non-modifiable factors that influence HCT readiness at the individual, family, and health care system levels during a pandemic like COVID-19 which impacted clinical care and family dynamics.^6^, ^7^, ^8^, ^11^ Factors like mental health of AYA and healthcare transition satisfaction may be impacted by the pandemic due to heightened anxiety and alterations to transition services (due to low staffing or divergence of resources) provided at healthcare facilities.^11^, ^41^

Thus, our study purpose was to develop an online survey to explore HCT readiness of AYA and modifiable and non-modifiable factors associated with HCT readiness among caregivers and their AYA with CF during COVID-19. For our analyses, we examined the distribution and potential association of healthcare factors at different levels of the AYA ecology to generate new insights that expand current HCT theories to improve research and practice. An adaptation of the Health Care Transition Research Consortium (HCTRC) Model and the Social-ecological Model of AYA Readiness for Transition (SMART) was used as a conceptual lens.^9^, ^10^ The HCTRC model provided a framework to assess individual (e.g., AYA demographics), family/social support (e.g., household structure), and health care system (e.g., HCT satisfaction) factors, while SMART provided guidance in assessing non-modifiable (e.g., demographics) and modifiable (e.g., caregiver resilience) factors that may be associated with HCT readiness.^9^, ^10^

**Fig. X:**
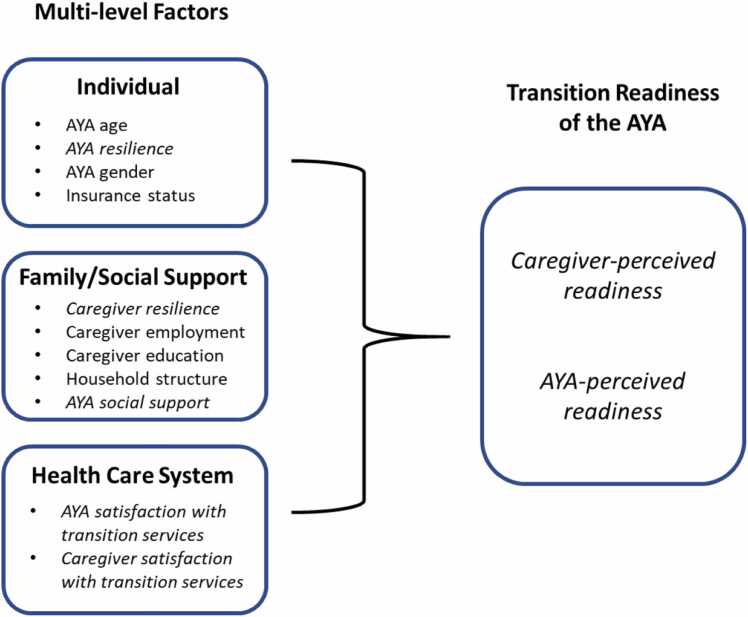
Adapted Conceptual Framework. *Note*. AYA is adolescents/young adults. Modifiable factors depicted in italics.

## 2 Methods

### 2.1 Design

A cross-sectional, multi-site, correlative design was used to explore perceived HCT readiness and associated factors among caregivers and their AYA with CF from three pediatric CF centers in the USA. Data were collected using an online Qualtrics survey; online surveys provide time for individuals to reflect and respond to questions, are low cost, and did not require in-person meetings during COVID-19.^12^ The Institutional Review Board (IRB) at the University of North Carolina at Chapel Hill and at each of the external sites approved this study.

### 2.2 Setting and sample

Caregivers and their AYA with CF were recruited from three large pediatric CF centers in Michigan, North Carolina, and Massachusetts; each center had their own HCT processes.^13^ The sampling goal was to recruit families (i.e., one caregiver and their AYA); to reduce risk of coercion, AYA participation was optional. Caregivers were included if they: (1) were a caregiver or legal guardian of an AYA aged 12–21 years with CF, (2) could speak and read English, and (3) provided an active email address to the CF center. AYA were included if they: (1) were diagnosed with CF and saw a pediatric care provider, (2) were 12 to 21 years old, (3) could speak and read English, and (4) had access to a caregiver-provided email address. AYA were excluded if they: (1) were only receiving palliative care, (2) received an organ transplant/under evaluation for transplant, or (3) had a care center documented cognitive impairment that would hinder their ability to complete an online survey.^13^

### 2.3 Recruitment and data collection

Guided by Dillman et al.,^12^ recruitment and data collection occurred during COVID-19 from February-November 2021. Notably, data were collected during a time of unparalleled stress in which healthcare facilities were overwhelmed by high patient volumes, concerns about infection control and individual safety, severe staffing shortages, and low available resources for patient care and personal safety.^40^ Emergency protocols impacted both inpatient and outpatient care and research capabilities were reduced. Despite these challenges, external CF center staff obtained verbal consent from eligible caregivers to share their email addresses with the primary investigator, who then emailed caregivers personalized study invites with a survey link. Caregivers who provided informed consent via the link received the survey. Caregivers could also provide permission for any of their eligible AYA to also participate (if AYA was 12–17 years), or they could provide an email address for their older AYA (aged 18–21 years) with the AYA’s permission. AYA were then emailed personalized invites with a survey link. Informed consent was obtained from older AYA (18–21 years), and informed assent was obtained from younger AYA (12–17 years). Caregivers of younger AYA and older AYA could provide optional HIPAA authorization. Up to five reminder emails were sent to all non-respondents and $10 gift cards were provided to completed respondents to decrease nonresponse bias.^12^ Recruitment ended after the limit of reminder emails was reached for all non-respondents.

Data were collected using the online Qualtrics survey platform with surveys designed for caregivers (65 items) and AYA (70 items). The surveys included open-ended items with text fields and closed-ended items with yes/no responses and other response formats (e.g., Likert scales). Survey items were chosen based on the HCTRC and the SMART theoretical models.^9^, ^10^ Permission from each developer was obtained to use the STAR_x_ Transition Readiness Questionnaire, Mind the Gap Scale, Multidimensional Scale of Perceived Social Support, and the Connor-Davidson Resilience Scale.^14^, ^15^, ^16^, ^17^ Validity and reliability of each measure is described in Table A in the Appendix. Preliminary feasibility and acceptability of our survey was determined among a separate group of 12 caregivers and four AYA with CF from one of our sites; a summary can be found in Table B in the Appendix. Here we report analysis of the closed-ended survey items; findings from analysis of the open-ended items are reported elsewhere.^18^

### 2.4 Measures

#### 2.4.1 Outcome variables

The primary outcomes were (a) caregiver-perceived HCT readiness of the AYA and (b) AYA-perceived HCT readiness. The parent (STAR_x_-P) and youth (STAR_x_-youth) research versions of the STAR_x_ Transition Readiness Questionnaire (13-items) were used.^19^ Item scores ranged from 1 (‘never’, ‘nothing’, or ‘very hard’) to 5 (‘always’, ‘a lot’, or ‘very easy’).^14^, ^19^ Subdomain scores included AYA disease knowledge (scores 0–20), self-management skills (scores 0–25), and provider communication skills (scores 0–20).^19^ Subdomain scores were added together for a total score ranging from 0–65; higher scores indicated greater perceived HCT readiness.^14^, ^19^

#### 2.4.2 Explanatory variables

Selection of explanatory variables was guided by the HCTRC model, while the SMART guided determination of the modifiability of variables.^9^, ^10^

Non-modifiable individual-level variables included: AYA age (<18 years; >18 years); AYA gender (male; female); and private insurance (public or private & public; private only). Non-modifiable family/social support-level variables included: household structure (less than two caregivers at home; two or more caregivers at home); caregiver education (less than a two-year college degree; at least a two-year college degree); and caregiver employment (part-time, unemployed or other; full-time).

Modifiable variables were also included. First, the Connor-Davidson Resilience scale (10 items) measured caregiver-reported resilience (family/social support-level) and AYA-reported resilience (individual-level); items ranged from 0 (‘not true at all’) to 4 (‘true nearly all the time’) and items were added to calculate the total score (0–40, higher scores indicated greater resilience).^15^ Second, the Multidimensional Scale of Perceived Social Support (12 items) was only given to AYA and measured AYA-perceived social support (family/social support-level); items ranged from 1 (‘very strongly disagree’) to 7 (‘very strongly agree’).^17^ Final scores were based on the average of the total score (score range of 1–7, higher scores indicated greater social support).^17^ Third, the Mind the Gap Scale measured caregiver and AYA satisfaction with AYA HCT services (healthcare system-level).^16^ Separate caregiver (27 items) and AYA versions (22 items) were used, and items ranged from 1 (‘strongly disagree’) to 7 (‘strongly agree’). Total scores were averaged across the gap scores (i.e., difference between ‘best’ and ‘current’ care) and ranged from −7 to 7; scores closer to −7 indicated higher satisfaction.^16^

#### 2.4.3 Chart data

For AYA with HIPAA authorization, data were abstracted from CF center electronic records to enhance sample characteristics and reduce self-report recall bias. Abstracted items are described in Table 1a, Table 1b based on literature from Lanzkron et al.^2^ and Suris & Akre.^20^ Abstracted items included body mass index, forced expiratory volume in one second, diagnoses of anxiety, depression, or CF-related diabetes, current feeding tube present, CF clinic attendance, hospitalizations, emergency room visits, and whether an AYA was prescribed a cystic fibrosis transmembrane conductance regulator modulator & duration of time on the modulator.

### 2.5 Data analysis

Survey data was exported from Qualtrics for data cleaning, then data were assessed using SAS 9.4 (Cary, NC). Internal consistency/reliability of each measure was assessed using Cronbach’s coefficient alpha.^29^ Descriptive statistics were used to characterize the sample and all key analytic variables. Non-directional statistical tests were performed with statistical significance set at 0.05 per test. Due to sample sizes and non-normality of the data distributions for continuous measures, a non-parametric approach was applied.

Spearman correlation coefficients were used to determine the associations between continuous factors and HCT readiness, while Wilcoxon Two-Sample tests were used to test associations between categorical factors and HCT readiness.^22^ A Wilcoxon Two-Sample test was also conducted to determine differences in HCT readiness across CF centers due to differing HCT procedures.^22^ An a priori power analysis using GPower3.1 indicated a sample size of 64 caregivers and 64 AYA were needed to achieve 80 % power for the testing of planned associations, assuming statistical significance at 0.05 and medium effect size (ρ) of 0.30.^21^ Analyses were exploratory and sought to identify trends, particularly in our AYA sample.

## 3 Results

### 3.1 Final analysis samples and measure reliability

A total of 392 caregivers were screened for study eligibility, and 234 were invited to participate. Among the 234 caregivers, 90 responded to our survey and 73 completed it. Among these 73 caregivers, 61 granted permission for their AYA to participate in our study. Of the 61 AYA invited, 37 responded and 34 completed the survey. Two of the 73 caregivers were omitted from final analysis due to missing demographics and unreliable data (e.g., responding ‘0′ to everything). One of the 34 AYA was omitted from final analysis due to missing data on two measures. Thus, the final analysis sample was *N* = 71 caregivers and *N* = 33 AYA.

Regarding internal consistency/reliability, the STAR_x_ total score had ‘very good’ (Cronbach *α* = 0.84) and ‘respectable’ (Cronbach *α* = 0.74) reliability among caregivers and AYA, respectively. All other measures had ‘respectable’ (Cronbach α = 0.79) to ‘very good’ (Cronbach α = 0.90) score reliability among caregivers and AYA.

### 3.2 Sample characteristics

Sample characteristics are described in Table 1a, Table 1b. Caregivers were predominately White (*n* = 70; 99 %), mothers (*n* = 61; 86 %), had at least a 2-year college degree (*n* = 62; 87 %), and worked full-time (*n* = 40; 56 %). One caregiver identified as Black or African American. One caregiver had two AYA complete our survey, 32 caregivers had one AYA complete, and 38 caregivers had no AYA complete our survey. Median caregiver resilience was 30.0 (range: 16–40), and the median caregiver transition services satisfaction was 0.0 (range: −1.1 to 2.6).

**Table 1a:** Sample Characteristics.

Characteristic	AYA (*N* = 33) *n (%)*	Caregiver (*N* = 71) *n (%)*
White race	29 (87.9)	70 (98.6)
Female gender	24 (72.7)	64 (90.1)
Caregiver type		
Mother		61 (85.9)
Father		7 (9.9)
Step-parent		2 (2.8)
Aunt		1 (1.4)
Multiple children with CF		16 (22.5)
Education level		
At least a 2-year college degree		62 (87.3)
Less than a 2-year college degree		9 (12.7)
Full-time employment		40 (56.3)
Two or more caregivers at home		51 (71.8)
AYA insurance		
Public	10 (30.3)	
Private	16 (48.5)	
Public & Private	7 (21.2)	
AYA regularly sees PCP	30 (90.9)	
**Clinical characteristics**	***N*****= 28**	
Anxiety diagnosis	13 (46.4)	
Depression diagnosis	3 (10.7)	
Current feeding tube	6 (21.4)	
CF-related diabetes	2 (7.1)	
Prescribed a CFTR modulator	26 (92.9)	
CF clinic visits (virtual/in-person in 2020)		
0 −1 visits	0 (0.0)	
2 −3 visits	9 (32.1)	
4 −5 visits	19 (67.9)	
Hospitalizations (in 2020)		
0 hospitalizations	25 (89.3)	
1 −5 hospitalizations	3 (10.7)	
Emergency visits (in 2020)		
0 visits	24 (85.7)	
1 visit	4 (14.3)	

*Note.* CF = cystic fibrosis; PCP = primary care provider; AYA = adolescents/young adults. Five of the 33 AYA did not have HIPAA authorization for review of clinical chart characteristics.

**Table 1b:** Sample Characteristics.

Characteristic	AYA (*N* = 33) *Median (25*^*th*^*, 75*^*th*^*)*	Caregiver (*N* = 71) *Median (25*^*th*^*, 75*^*th*^*)*
AYA age, in years	15.0 (14.0, 17.0)	
**Clinical characteristics**	***N*****= 28**	
BMI, value	21.0 (20.2, 23.6)	
BMI, percentile (*n* = 26)	67.1 (44.0, 82.8)	
FEV_1_, average % in 2020	99.5 (87.2, 107.0)	
Duration on CFTR modulator, in years (*n* = 24)	2.0 (1.5, 3.5)	
**Survey scale scores**		
Resilience of the respondent	28.0 (23.0, 32.0)	30.0 (26.0, 35.0)
AYA perceived social support	6.1 (5.2, 6.8)	
Satisfaction with AYA transition services	0.0 (−0.5, 1.1)	0.0 (−0.3, 0.6)
**Healthcare transition readiness scores**		
AYA-perceived transition readiness	51 (48, 55)	
Caregiver-perceived transition readiness		50 (45, 56)

*Note.* BMI = body mass index; FEV_1_ = forced expiratory volume in one second; CFTR = cystic fibrosis transmembrane conductance regulator. Resilience of the respondent = Connor-Davidson resilience total score (range: 0-40), higher scores indicate greater resilience; AYA’s perceived social support = Multidimensional Scale of Perceived Social Support average score (range: 1-7), higher scores indicate greater social support; Satisfaction with the AYA’s transitional care services = Mind the Gap Scale average score (range: −7 to 7), scores closer to −7 indicate higher satisfaction, scores of 0 indicate no difference between ‘best’ and ‘current’ care, and scores closer to 7 indicate lower satisfaction; Healthcare transition readiness of the AYA = STAR_x_ total score (range: 0-65), higher scores indicate greater transition readiness. Five of the 33 AYA did not have HIPAA authorization for review of clinical characteristics; two of the 28 AYA did not have BMI percentile assessed per care center policy; two of the 26 AYA prescribed a CFTR modulator did not have data on duration of time on modulator.

Most AYA were White (*n* = 29; 88 %); two AYA (6 %) identified as Black or African American, and two as multiracial (6 %). Most AYA were female (*n* = 24; 73 %); two AYA (6 %) identified as transgender. The median AYA age was 15 years (range: 12–20). The median BMI value was 21.0 (range: 16.2–31.9), and the median FEV_1_ was 99.5 (range: 53.6–124.0). 13 AYA (46 %) had an anxiety diagnosis, and 3 AYA (10 %) had a depression diagnosis. Additionally, the median AYA resilience score was 28.0 (range: 16–38), the median social support score was 6.1 (range: 4.3–7.0), and the median AYA satisfaction with transition services score was 0.0 (range: −0.9 to 2.5).

### 3.3 Healthcare transition readiness outcomes

As shown in Table 1b, the median caregiver-perceived HCT readiness score was 50 (range: 32 to 63). The median AYA-perceived HCT readiness score was 51 (range: 34 to 62). Caregiver- and AYA-perceived HCT readiness was not statistically different across the three sites (all *p* > 0.05).

### 3.4 Modifiable Characteristics Associated with Healthcare Transition Readiness

From Table 2, there was a significant, moderate correlation between caregiver resilience and caregiver-perceived HCT readiness (*r*_*s*_ = 0.32, *p* = 0.0061).

**Table 2:** Relationships between Modifiable Characteristics with AYA Transition Readiness.

**Modifiable Characteristics**	**AYA-perceived** Transition Readiness STAR_x_ Total Score (*N* = 33)	**Caregiver-perceived** Transition Readiness STAR_x_ Total Score (*N* = 71)
AYA resilience score	0.14	-
AYA social support score	0.21	-
AYA satisfaction with transition services score	−0.07	-
Caregiver resilience score	-	0.32 * *
Caregiver satisfaction with transition services score	-	−0.21

*Note.* Spearman correlation coefficients, * * *p* = 0.0061, all remaining correlations had *p* > 0.05. The satisfaction measure is scaled such that negative values indicate higher satisfaction. Effect size cut offs: *r* = 0.10, small; *r* = 0.30, medium; *r* = 0.50, large.

### 3.5 Non-modifiable characteristics associated with healthcare transition readiness

As described in Table 3, there were no statistically significant relationships between caregiver- or AYA-perceived HCT readiness and any non-modifiable factors (all *p* > 0.05). Although not statistically significant, AYA males tended to self-report lower HCT readiness compared to females (*p* = 0.0719).

**Table 3:** Relationships between Non-Modifiable Characteristics with AYA Transition Readiness.

**Non-Modifiable Characteristics**	***n***	**AYA-perceived Transition Readiness***Median (25*^*th*^*, 75*^*th*^*)*	***p***
AYA ≥ 18 years old AYA < 18 years old	7 26	53.0 (50.0, 58.0) 51.0 (46.0, 55.0)	0.2166
AYA Female AYA Male	24 7	52.5 (49.5, 55.5) 41.0 (38.0, 55.0)	0.0719
AYA has private insurance only AYA public insurance or private and public	16 17	51.5 (49.0, 55.0) 51.0 (47.0, 55.0)	0.9281
Caregiver full-time employment Caregiver part-time work; unemployed; other	20 13	51.0 (48.0, 56.0) 52.0 (48.0, 53.0)	0.9118
Caregiver with at least a 2-year college degree Caregiver with less than 2-year college degree	27 6	51.0 (47.0, 55.0) 54.0 (49.0, 56.0)	0.4265
Caregiver - two or more caregivers at home Caregiver - less than 2 caregivers at home	23 10	52.0 (47.0, 56.0) 50.5 (50.0, 53.0)	0.7386
**Non-Modifiable Characteristics**	***n***	**Caregiver-perceived Transition Readiness***Median (25*^*th*^*, 75*^*th*^*)*	***p***
AYA ≥ 18 years old AYA < 18 years old	24 47	52.5 (46.0, 59.0) 50.0 (44.0, 54.0)	0.0861
AYA Female AYA Male	41 26	52.0 (46.0, 57.0) 49.0 (45.0, 52.0)	0.1700
AYA has private insurance only AYA public insurance or private and public	42 29	50.5 (47.0, 56.0) 50.0 (42.0, 54.0)	0.3578
Caregiver full-time employment Caregiver part-time work; unemployed; other	40 31	50.5 (46.0, 58.0) 48.0 (43.0, 53.0)	0.1567
Caregiver with at least a 2-year college degree Caregiver with less than 2-year college degree	62 9	50.0 (45.0, 56.0) 52.0 (43.0, 54.0)	1.0000
Caregiver - two or more caregivers at home Caregiver - less than 2 caregivers at home	51 20	51.0 (46.0, 56.0) 48.0 (41.0, 53.0)	0.1463

*Note*. Wilcoxon Two-Sample test performed.

## 4 Discussion

In this study of caregiver- and AYA-perceived HCT readiness of youth with CF, we observed moderately high readiness for HCT and found that caregiver resilience was correlated with caregiver-perceived HCT readiness of AYA.

Our AYA sample was relatively healthy with BMI in optimal range for CF (>21 females; >22 males), normal lung function (percent predicted FEV_1_ >80 %), and few hospitalizations.^2^, ^3^ Most AYA (*n* = 30; 91 %) reported they regularly visited a primary care provider (PCP) who was different from their specialist. This finding contrasts with the ∼9.2 million U.S. youth with special healthcare needs who reported not having a medical home in 2022.^1^ PCPs can provide a key medical home during the transition process and it may be that the multisystem involvement in CF enhances engagement with PCPs.^2^ Moreover, 13 AYA had a documented anxiety diagnosis and may have been seeing PCPs, who are typically closer in distance compared to CF care centers, for comprehensive care services especially with the rise of mental health challenges during COVID-19.^11^

Caregivers and AYA similarly perceived a moderately high HCT readiness of AYA for transitions to adult-focused care. A comparable study using the STAR_x_ measure reported slightly lower HCT readiness scores, with caregivers reporting lower perceived HCT readiness than their AYA.^19^ It may be that COVID-19 healthcare changes (e.g., telehealth visits) promoted AYA readiness in our study via enhanced one-on-one provider communication.^18^ However, due to use of different measures of HCT readiness in other studies, it is difficult to compare readiness scores across studies or during COVID-19 when HCT processes were altered.^6^ In our study, male AYA tended to report lower readiness compared to females which aligns with prior literature; additional research with larger male samples should be sought to confirm this finding.^6^ Interestingly, AYA age was not significantly associated with perceived HCT readiness. This may be due to our smaller sample of AYA ≥ 18 years who may have been harder to reach due to competing interests (e.g., higher education pursuits, employment, individual and/or family responsibilities). Future research should develop tailored recruitment materials to enhance interest and participation among these older AYA.

We found caregiver resilience, defined by Connor & Davidson^15^ as the ability to thrive in adversity, was a family-level modifiable factor correlated with caregiver-perceived HCT readiness of AYA. Families dealing with chronic conditions face many difficulties related to the health of AYA, and it may be that caregivers who overcome adversities by following healthcare provider guidance instill that same dedication to self-care management in their AYA, thus enhancing their perception (and confidence) in AYA HCT readiness skills.^28^ Findings from Hart et al.^26^ indicated that higher caretaker role demands are associated with lower caregiver-perceived readiness, suggesting caregiver resilience and the degree that AYA self-manage their care may be important drivers of caregiver-perceived HCT readiness of AYA. Confounding variables may influence the relationship between caregiver resilience and caregiver-perceived HCT readiness of AYA, indicating a need for a larger, more diverse sample to control for potential confounding variables and to confirm this finding. Once confirmed, existing resources from the Cystic Fibrosis Foundation could be utilized to fund HCT-focused interdisciplinary subteams (e.g., nurses, physicians, nutritionists, social workers, therapists) within each CF care center to co-develop and pilot test a family toolkit with available resources and local services aimed at promoting caregiver resilience and HCT readiness of AYA with CF.

Exploring caregiver resilience is a critical research priority determined by youth dealing with chronic conditions, their caregivers, and their healthcare providers.^38^ Despite this need, few studies and limited transition-specific measures assess caregiver resilience and how it may impact healthcare transition readiness of AYA, especially during the COVID-19 pandemic when healthcare changes have impacted care and outcomes.^11^, ^28^, ^35^, ^36^ Moreover, mental health issues increased during the pandemic, impacting families of AYA with CF who already experience an elevated rate of depression and anxiety.^4^, ^37^ Findings from related studies assessing resilience in families of young adult stroke patients indicate that family resilience impacts patient-reported outcomes like mental well-being.^39^ Our study examines this key research priority and expands prior research by highlighting the association between caregiver resilience and caregiver-perceived transition readiness of AYA with CF during COVID-19. Our findings provide preliminary evidence to explore a future strength-based, interdisciplinary, family toolkit to enhance resilience and patient-reported outcomes in this population.

### 4.1 Limitations

This study is not without limitations. Our final sample sizes were small and relatively homogenous and indicate a need for more studies with larger samples to confirm our significant findings. We used a cross-sectional design so we were unable to determine causal relationships with modifiable and non-modifiable factors that may influence HCT readiness. We used a convenience sample of recruitment sites which increases the risk for sampling bias.^12^, ^42^ As most of our sample was White and relatively healthy, the generalizability of our results are restricted to groups that share these same characteristics, and thus the external validity of our results may not accurately represent caregivers and AYA with CF in the US.^12^, ^42^ COVID-19 also had known and unknown influence on HCT readiness.^11^, ^41^ Due to low reliability of subdomain scores in our sample, we only used the STAR_x_ total score and thus were limited to perceptions of overall HCT readiness.^19^

### 4.2 Research Implications

**Fig. 1:**
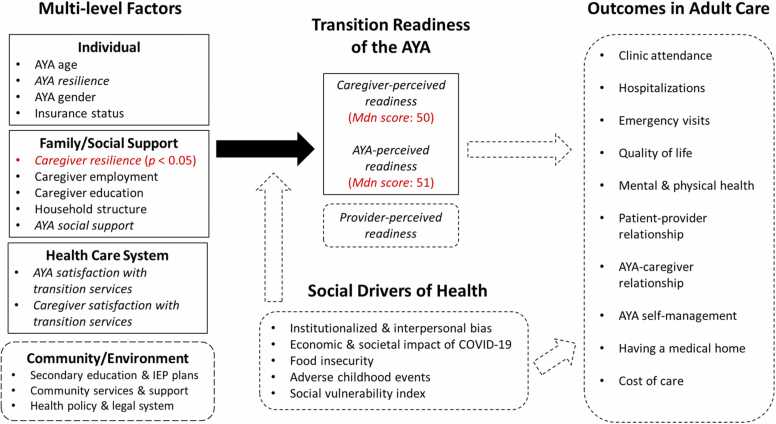
Concept Map of Findings and Future Research Directions. *Note*. AYA is adolescents/young adults; IEP is individualized educational program. Modifiable factors depicted in italics. Future research directions depicted by dashed boxes and arrows. Outcomes in adult care derived from post-healthcare transition outcomes described by Suris and Akre,^20^ Fair et al.23, and Berwick et al.27.

Despite these limitations, our study findings indicate a critical need to expand current HCT theories to guide future family research (Figure 1). Studies utilizing larger, more diverse samples of families are needed to identify modifiable multi-level factors depicted in the figure that may impact HCT readiness.^9^, ^10^ Hierarchical regression could identify whether HCT readiness is most impacted by individual, family, healthcare, or community-level factors above all other factors.^22^ Future studies should assess provider-perceived HCT readiness to measure the degree of agreement between caregivers, AYA, and their providers. Gaps in knowledge also exist surrounding the impact of social drivers of health (SDOH) on HCT readiness and future adult outcomes.^24^, ^25^ Early research indicates SDOH like poverty are associated with family factors and higher odds of poor transitional care, thus SDOH may moderate the relationships between multi-level factors and readiness.^24^ Theoretical models also describe SDOH as directly causing inequities in health outcomes.^25^ Structural equation modeling is therefore needed to better understand the theoretical relationships depicted in Figure 1.^22^ Future studies should additionally employ longitudinal designs to assess the associations between multi-level factors on HCT readiness, and HCT readiness on key outcomes in adult-focused care like patient-provider relationships.^20^, ^23^, ^27^

## 5 Conclusion

Caregivers and AYA shared perceptions of moderately high levels of AYA HCT readiness for a future transition to adult-focused care, and caregiver resilience was a key correlate of caregiver-perceived HCT readiness of AYA. Future research should replicate this study with a larger, more diverse sample of families to provide evidence for the development of an interdisciplinary toolkit aimed at enhancing family resilience and AYA readiness for adult-focused care. Future studies should also determine community-level factors and social drivers of health that impact caregiver-, AYA-, and provider-perceived HCT readiness of AYA and how these factors may be associated with key clinical outcomes in adult care.

## Ethical Approval

All study activities were approved by the Institutional Review Board at the University of North Carolina at Chapel Hill, and the Institutional Review Board at each of the collaborating sites (University of Michigan and Boston Children’s Hospital).

## Funding Sources

This work was supported by the Robert Wood Johnson Foundation Future of Nursing Scholars Program [10.13039/100006808University of North Carolina at Chapel Hill]; and a Cystic Fibrosis Foundation Student Traineeship Award [GIRDWO20H0]. The funding sponsors had no role in the design, methods, subject recruitment, data collections, analysis, or preparation of this paper.

## CRediT authorship contribution statement

**Jennifer L Goralski:** Writing – review & editing, Resources, Conceptualization. **Susan G Silva:** Writing – review & editing, Visualization, Formal analysis. **Kirsten Kainz:** Writing – review & editing, Supervision, Conceptualization. **Tyra Claire Girdwood:** Writing – review & editing, Writing – original draft, Visualization, Validation, Supervision, Software, Project administration, Methodology, Investigation, Funding acquisition, Formal analysis, Data curation, Conceptualization. **Mark P Toles:** Writing – review & editing, Writing – original draft, Validation, Supervision, Project administration, Formal analysis, Conceptualization. **Ahmet Uluer:** Writing – review & editing, Resources. **Samya Z Nasr:** Writing – review & editing, Resources. **Elisabeth P Dellon:** Writing – review & editing, Resources. **Mary R Lynn:** Writing – review & editing, Conceptualization. **Maria E Díaz-González de Ferris:** Writing – review & editing, Resources, Conceptualization.

## Declaration of Competing Interest

The authors declare the following financial interests/personal relationships which may be considered as potential competing interests: Tyra Girdwood reports financial support was provided by the Robert Wood Johnson Foundation Future of Nursing Scholars Program. Jennifer Goralski reports research funding and speaker honoraria from the Cystic Fibrosis Foundation.

## Figures and Tables

**Table:** **Table A.** Validity and Reliability of Included Measures.

Measure	Validity	Reliability
STAR_x_ Transition Readiness Questionnaire (13-item research version)	Prior studies have reported the STAR_x_ has concurrent, predictive, and discriminant validity with caregivers and their AYA with chronic conditions^14^, ^31^	The internal consistency of the three subscales of the 13-item version were: disease knowledge (0.76, caregivers; 0.76, AYA), self-management (0.55, caregivers; 0.73, AYA), and provider communication (0.70, caregivers; 0.69, AYA)^19^
Connor-Davidson Resilience Scale (10-item)	Prior studies reported content validity among adolescents (aged 12 −15 years), young adults (mean age 18.8 years, SD 2.2), and caregivers of children with a chronic condition; it also had reported convergent and discriminant validity^15^, ^32^, ^33^, ^34^	The internal consistency of the entire scale was reported as α = 0.89; item-total correlations were reported as r = 0.30 −0.7015
Multidimensional Scale of Perceived Social Support (12-item)	The measure has reported content validity in youth (ages 11 −23 years) and construct validity^17^, ^30^	Internal consistency of the full scale was reported as *α* = 0.88, and the internal consistency for each subscale were reported as *α* = 0.91 (significant other/person), *α* = 0.87 (family), and *α* = 0.85 (friends)^17^
Mind the Gap Scale: caregiver version (27 items) & AYA version (22 items)	Shaw et al.^16^ reported face validity & content validity among caregivers and their AYA with JIA (juvenile idiopathic arthritis)	The internal consistency for the entire scale was reported as *α* = 0.94 (caregiver version) and *α* = 0.91 (AYA version)^16^

*Note.* AYA is adolescents/young adults.

**Table:** **Table B.** Preliminary Feasibility and Acceptability of our Online Survey.

	Feasibility	Acceptability
**Caregivers** (*n* = 12)	18 of 98 (18.40%) caregivers responded to our invitation12 of 18 (66.67%) caregivers completed the survey8 out of 9 (89%) caregivers who had an AYA aged 12 −17 years allowed their AYA to participateThe median survey completion time for caregivers was 41.5 min (IQR = 49, including outliers of 142 min and 171 h)Missing data:1 missing response about caregiver education2 caregivers (16.7%) had missing responses to the Mind the Gap Scale, 1 caregiver (8.3%) had missing responses to the Connor-Davison Resilience Scale, and 1 caregiver (8.3%) had missing responses to the STARxFor the open-ended questions: 16.7% caregivers did not answer a question about perceptions using apps, the CF RISE program, or virtual visits to get ready for adult-focused careNo caregiver-reported barriers to accessing the survey in Qualtrics; no email distribution errors (e.g., undelivered, duplicate emails) reported in the Qualtrics mailer system	• 10 caregivers (83.3%) reported the survey was easy to complete • 1 caregiver suggested the STAR_x_ measure should include the word ‘independently’ • 1 caregiver suggested one open-ended question (i.e., participants’ perceptions using apps, the CF RISE program, or virtual visits) should be broken into subtopics • In assessing whether the participants experienced anything about the HCT process not asked in our survey, 9 caregivers (75%) responded ‘no’ or ‘N/A′, and 1 caregiver suggested to have a question about transitions at home • In assessing if anything could improve the survey, 6 caregivers (50%) responded ‘nothing’ or ‘N/A,’ 1 caregiver suggested shortening the survey, and one caregiver suggested including a progress counter
**AYA** (*n* = 4)	• 5 of 10 (50%) AYA responded to our invitation • 4 of 5 (80%) AYA completed the survey • The median survey completion time for AYA was 11.5 min (*IQR* = 61, including an outlier of 130 min) • Missing data: o No missing data for the measures o For the open-ended questions: 50% of AYA did not answer a question about perceptions using apps, the CF RISE program, or virtual visits o 50% of AYA had missing data on a question that asked when they’d feel ready to move to adult-focused care • No AYA-reported barriers to accessing the survey in Qualtrics; no email distribution errors (e.g., undelivered, duplicate emails) reported in the Qualtrics mailer system	• All 4 AYA stated the survey was easy or not difficult to complete • No AYA suggested changes to the survey • In assessing whether the participants experienced anything about the HCT process that was not asked in our survey, all 4 AYA responded ‘no’ or ‘not really’ • In assessing if anything could improve the survey, 3 AYA (75%) responded ‘nothing’ or ‘N/A′

*Note*. AYA is adolescents/young adults.

**Table:** **Table C.** STROBE Statement: Items included in reports of cross-sectional studies.

	Item No	Recommendation	Page No
**Title and abstract**	1	(*a*) Indicate the study’s design with a commonly used term in the title or the abstract	Title page
		(*b*) Provide in the abstract an informative and balanced summary of what was done and what was found	1
Introduction			
Background/rationale	2	Explain the scientific background and rationale for the investigation being reported	2 −3
Objectives	3	State specific objectives, including any hypotheses	2 −3
Methods			
Study design	4	Present key elements of study design early in the paper	3
Setting	5	Describe the setting, locations, and relevant dates, including periods of recruitment, exposure, follow-up, data collection	3 −4
Participants	6	(*a*) Give the eligibility criteria, and the sources and methods of selection of participants	3 −4
Variables	7	Clearly define all outcomes, exposures, predictors, potential confounders, and effect modifiers. Give diagnostic criteria	5 −6
Data sources/ measurement	8	For each variable of interest, give sources of data and details of methods of assessment. Describe comparability of assessment methods if there is more than one group	5 −6
Bias	9	Describe any efforts to address potential sources of bias	6 −7
Study size	10	Explain how the study size was arrived at	7
Quantitative variables	11	Explain how quantitative variables were handled in the analyses. If applicable, describe which groupings were chosen and why	6 −7
Statistical methods	12	(*a*) Describe all statistical methods, including those used to control for confounding	6 −7
		(*b*) Describe any methods used to examine subgroups and interactions	N/A
		(*c*) Explain how missing data were addressed	7
		(*d*) If applicable, describe analytical methods taking account of sampling strategy	6 −7
		(*e*) Describe any sensitivity analyses	N/A
Results			
Participants	13	(a) Report numbers of individuals at each stage of study—eg numbers potentially eligible, examined for eligibility, confirmed eligible, included in the study, completing follow-up, and analyzed	7
		(b) Give reasons for non-participation at each stage	7
		(c) Consider use of a flow diagram	-
Descriptive data	14	(a) Give characteristics of study participants (eg demographic, clinical, social) and information on exposures and potential confounders	7 −8
		(b) Indicate number of participants with missing data for each variable of interest	7
Outcome data	15	Report numbers of outcome events or summary measures	8
Main results	16	(*a*) Give unadjusted estimates and, if applicable, confounder-adjusted estimates and their precision (eg, 95% confidence interval). Make clear which confounders were adjusted for and why they were included	8
		(*b*) Report category boundaries when continuous variables were categorized	N/A
		(*c*) If relevant, consider translating estimates of relative risk into absolute risk for a meaningful time period	N/A
Other analyses	17	Report other analyses done—eg analyses of subgroups and interactions, and sensitivity analyses	N/A
Discussion			
Key results	18	Summarize key results with reference to study objectives	9 −10
Limitations	19	Discuss limitations of the study, taking into account sources of potential bias or imprecision. Discuss both direction and magnitude of any potential bias	10 −11
Interpretation	20	Give a cautious overall interpretation of results considering objectives, limitations, multiplicity of analyses, results from similar studies, and other relevant evidence	9 −12
Generalizability	21	Discuss generalizability (external validity) of the study results	9 −12
Funding	22	Give the source of funding and the role of the funders for the present study and, if applicable, for the original study on which the present article is based	Title page

## Data Availability

The data that has been used is confidential.
